# Atrial Fibrillation in Older People: Concepts and Controversies

**DOI:** 10.3389/fmed.2019.00175

**Published:** 2019-08-08

**Authors:** Zafraan Zathar, Anne Karunatilleke, Ameenathul M. Fawzy, Gregory Y. H. Lip

**Affiliations:** ^1^Institute of Applied Health Research, University of Birmingham, Birmingham, United Kingdom; ^2^Liverpool Centre for Cardiovascular Science, University of Liverpool and Liverpool Heart and Chest Hospital, Liverpool, United Kingdom; ^3^Aalborg Thrombosis Research Unit, Department of Clinical Medicine, Aalborg University, Aalborg, Denmark

**Keywords:** atrial fibrillation, older people, elderly, management, oral anti-coagulation, stroke, frailty, cognitive impairment

## Abstract

Atrial fibrillation (AF) is the commonest cardiac rhythm abnormality and has a significant disease burden. Amongst its devastating complications is stroke, the risk of which increases with age. The stroke risk in an older person with AF is therefore tremendous, and oral-anticoagulation (OAC) therapy is central to minimizing this risk. The presence of age-associated factors such as frailty and multi-morbidities add complexity to OAC prescription decisions in older patients and often, OAC is needlessly withheld from them despite a lack of evidence to support this practice. Generally, this is driven by an over-estimation of the bleeding risk. This review article provides an overview of the concepts and controversies in managing AF in older people, with respect to the existing evidence and current practice. A literature search was conducted on Pubmed and Cochrane using keywords, and relevant articles published by the 1st of May 2019 were included. The article will shed light on common misconceptions that appear to serve as rationale for precluding OAC and focus on clinical considerations that may aid OAC prescription decisions where appropriate, to optimize AF management using an integrated, multi-disciplinary care approach. This is crucial for all patients, particularly older individuals who are most vulnerable to the deleterious consequences of this condition.

## Introduction

Atrial fibrillation (AF) is the commonest cardiac rhythm disturbance affecting the general population. In 2010, the number of individuals with AF in the European Union over the age of 55 years was estimated to be 8.8 million but this is expected to double to 17.9 million by 2060, with a further 6–12 million likely to be affected in the United States (US) (1, 2). It is a significant public health burden estimated to cost the UK National Health Service (NHS) and US healthcare £459 million and up to $26 billion, respectively (3, 4).

Although asymptomatic in the vast majority of patients, AF can manifest as ischaemic strokes, leading to hospitalisations, and death. Indeed, AF is associated with a 5-fold increase in the stroke risk (5). Its prevalence in the general population increases steadily with advancing age, from 0.12–0.16% in people younger than 49 years to 3.7–4.2% in those aged 60–70 years. Beyond the age of 80 years, prevalence can be as high as 10–17% (6).

As age is an independent risk factor for AF, the global burden of this condition is expected to increase with the aging population. However, management and treatment of this common arrhythmia in older people has proven to be a dilemma for many, as they are a heterogeneous group of individuals complicated by the presence of functional, and social factors that contribute to their vulnerability, in addition to multimorbidity and polypharmacy; all of which need to be taken into account. It is also partially attributable to the lack of guidelines specific to this patient population.

We aim to provide an overview of the concepts and controversies in managing AF in older individuals, with respect to current practice, and existing evidence. A literature search was conducted on Pubmed and Cochrane databases using the keywords “atrial fibrillation,” “oral anti-coagulation,” “management,” “older,” “elderly,” “falls” “frailty,” and “cognitive impairment.” Relevant articles published by the 1st of May 2019 were included. Randomized controlled trials (RCTs), systematic reviews and meta-analyses are presented as they provide a higher quality of evidence. Other studies including registry studies and observational studies are also included, as they make-up a significant proportion of the evidence base for this population, given the lack of large-scale RCTs, and provide a more “real-world” perspective on oral anti-coagulation (OAC) use in older patients. When selecting relevant papers, the overall study design, and corresponding results were taken into consideration.

## AGING and Atrial Fibrillation

The relationship between AF and age has been well-described in the literature and epidemiological studies have been instrumental in establishing this link. Indeed, advancing age is the most prominent risk factor for AF. In the Framingham cohort study which followed individuals over a 22 year period, the incidence of AF was noted to increase with advancing age. Age, along with hypertension, congestive heart failure, diabetes mellitus, coronary artery disease, and valvular disease were identified as independent risk factors for the development of AF (7, 8). This was followed by further studies confirming the link between age and AF, which is now well-established (9–14).

Age is also an independent risk factor for stroke (15). This was signified in the landmark Atrial Fibrillation Investigators (AFI) schema which identified age as a predictor of stroke in AF patients (16). The Stroke Prevention in AF (SPAF) study also recognized its pertinence, noting that females over 75 years had a higher rate of thromboembolic events (17). The age-associated increase in the risk of stroke is not specific to sex and is observed both in males and females (18–21). In the Framingham study, stroke risk increased significantly from 1.5 to 23.5% at 50–59 years, and 80–89 years respectively, and in the latter age group, AF was the sole cardiovascular condition to exert an independent effect on stroke incidence (10).

Age is not a static but a dynamic risk factor for AF-related stroke, and risk increases from 65 years and upwards in Western populations (22). It is a continuous variable, making it difficult to establish an arbitrary cut-off for risk stratification purposes. Table 1 outlines the current version of the CHA_2_DS_2_-VASc risk stratification tool which is most widely used in clinical practice.

**Table 1:** Stroke risk stratification: CHA_2_DS_2_-VASc score.

**Risk factor**	**Score**
Congestive heart failure	1
Hypertension	1
Age ≥75 years	2
Diabetes mellitus	1
Stroke/TIA	2
Vascular disease (prior MI,PAD or aortic plaque)	1
Age 65–74 years	1
Sex category: Female	1

*The CHA_2_DS_2_-VASc score calculates stroke risk in atrial fibrillation. Score of 0 is “low risk” may not require anticoagulation. Score of 1 in males and ≥2 in females is “low-moderate” risk and anti-coagulation may be considered as per ESC guidelines. A score of ≥2 and ≥3 in males and females respectively, is “moderate-high” risk and anticoagulation is indicated*.

*Maximum possible score = 9; TIA-transient ischemic attack; MI-myocardial infarction; PAD-peripheral arterial disease*.

## Pathophysiology of AF in Older Individuals

The numerous risk factors that increase the propensity for AF can be grouped into modifiable or non-modifiable risk factors (23). Modifiable risk factors include body mass index (BMI), diabetes, obstructive sleep apnoea (OSA) and hypertension whereas the non-modifiable risk factors include genetics, gender, ethnicity, and of course, age (24). These increase susceptibility to AF by inducing structural and histopathological changes.

The onset of AF requires both an initiating trigger and an anatomical substrate; in this case, a critical mass of abnormal tissue that can provoke atrial ectopic beats and give rise to paroxysms of AF. The pulmonary veins (PVs) may be the main origin for ectopic triggering foci (25, 26). Nevertheless, the exact pathophysiological mechanisms linking age and AF are poorly understood. In older patients, the presence of multiple comorbidities adds to the complexity of establishing the impact of aging vs. the impact of comorbidities on the development of AF, in isolation. The aging heart offers an ideal environment for AF to flourish in the presence of predisposing “anatomical substrate” abnormalities, due to conditions such as hypertension, ischaemic heart disease, heart failure, valvular disease, and dilated/hypertrophic cardiomyopathy (27, 28). These have been associated with histopathological and atrial chamber abnormalities which result in myocardial fibrosis and atrial dilation, thus increasing the risk of AF (29). In addition, the stretching of the atrial fibers due to atrial enlargement leads to a shorter refractory period and slower electrical conduction, further optimizing the conditions for arrhythmogenicity (27).

Over time, repeated episodes of AF lead to further substrate abnormality through structural and electrophysiological remodeling, and modify the cell to cell conduction, resulting in a reduced threshold for AF triggers and conditions which sustain AF (30). AF therefore gives rise to additional risk factors which contribute to its own progression; hence, “AF begets AF,” explaining how paroxysmal episodes progress into permanent sustained episodes over a period of time. The longer the treatment is delayed, the more difficult it is to regain sinus rhythm (27).

## Anticoagulation in Older People

Stroke risk is not homogeneous and risk factors have been used to formulate various stroke (and bleeding) risk stratification schemes (31). Risk is not a static “one off” process but is dynamic in nature, and the change in risk profile leads to an increased risk for outcomes (32, 33).

Both the European Society of Cardiology (ESC) and National Institute for Health and Care Excellence (NICE) guidelines (UK) recommend assessment of stroke risk using the CHA_2_DS_2_-VASc score with view to considering OAC for scores of ≥1 in males and ≥2 in females (34, 35). OAC with a vitamin K antagonist (VKA) or the newer non-vitamin K antagonist oral anti-coagulants (NOACs) must be recommended for those who meet the criteria. The guidelines are in agreement that OACs are superior to aspirin and aspirin monotherapy should not be offered to patients with AF, solely for stroke prevention. The American College of Cardiology/American Heart Association/Heart Rhythm Society (ACC/AHA/HRS) guidelines differ slightly with OAC being recommended for higher CHA_2_DS_2_-VASc scores of ≥2 and ≥3 in men and women, respectively. For scores of 1, management options include withholding OAC, or treatment either with an OAC or aspirin (36).

Given that the default is to offer stroke prevention unless patients are “low risk,” the recommendations have shifted to initially identify low risk patients first (rather than high risk ones) who do not need any antithrombotic therapy (37). The next step is to offer stroke prevention to those with ≥1 additional stroke risk factors (38).

### Perceptions Regarding Oral Anti-coagulation

As previously established, both age and AF are independent risk factors for stroke, meaning that the older patient with AF is particularly vulnerable to developing a stroke (15). The meta-analysis by Albertsen et al. (39) showed that older patients are more likely to have a stroke even when on an OAC, despite a 64% stroke risk reduction with warfarin (40). Indeed, this suggests that there is greater benefit to be derived from OACs by those who are older. Despite this, OAC underuse remains a pressing issue in the older patient population (41).

Pugh et al. (42) in a systematic review explored physicians' attitudes toward OAC for stroke prevention in older people. Barriers to OAC prescription in order of most cited reasons included bleeding risk, falls risk, age, and patients' ability to comply with the treatment regimen. The study also reported that even in the absence of any contraindications to warfarin, physicians were still unlikely to recommend OAC for patients over 70 compared to those ≤70 years of age. In another review, limited evidence or perceived uncertainty, the need for individualized decision making and feelings of delegated responsibility were identified as key physician concerns which deterred them from prescribing OAC (43).

These reservations by clinicians are partially justified by the limited evidence specific to this cohort. Few RCTs specific to older people have been carried out and even then, older patients are under-represented in the existing RCTs. In most cases, results from such studies are extended to these patients and their direct applicability to the older individual is frequently questioned.

In addition, the CHA_2_DS_2_-VASc score which is largely driven by age is poorly helpful in determining the therapeutic approach in older individuals. This is particularly true for those over 75 years, for whom OAC therapy is indicated by definition, regardless of the presence of other factors. In essence, this makes the tool almost unnecessary in cardio-geriatric practice and what would be more helpful is a decision-making guide that incorporates factors such as frailty, and comorbidities such as dementia which influence treatment decisions in this age group. This is also true for the bleeding risk scoring systems, most of which incorporate age, and stroke. Thus, the overlap in these components of the risk stratification scores result in a parallel increase in the stroke and bleeding risks that is less helpful and to a certain extent, these scores almost seem like an oxymoron.

Indeed, risk stratification scores serve as tools to guide treatment decisions and must not be used as replacements for existing evidence and/or clinical judgement, i.e., bleeding risk scores should be used to identify patients who are at a higher risk of bleeding, to initiate closer monitoring strategies as advocated by the guidelines (34, 36). Yet, they are often used to justify withholding OAC therapy from patients who have an equal or higher risk of stroke (44). In their study, Friberg et al. (45), demonstrated that the risk of ischaemic stroke without OAC was higher than the risk of bleeding with OAC, unless the risk of ischaemic stroke was very low (CHA_2_DS_2_-VASc = 0). For a patient with a CHA_2_DS_2_-VASc and HAS-BLED (comprising of hypertension, renal impairment, liver dysfunction, stroke history, prior major bleeding, or predisposition to bleeding, labile INR, age >65 years, medications predisposing to bleeding and drug/alcohol use) score of 5, the net clinical benefit (NCB) of OAC was still 3% per year, even with a weight of 1.5 applied to intra-cranial hemorrhage (ICH) events, to account for their disastrous consequences.

Nonetheless, decision-making aids that are more specific to older people, inclusive of age-associated factors, may help minimize some of the physician concerns and uncertainties toward prescribing OAC.

### Conventional Oral Anticoagulants

In the general population, it is well-established that warfarin is superior to both mono and dual anti-platelet therapy. In a systematic review and network meta-analysis, López-López et al. (46) demonstrated that aspirin <150 mg once daily [OR 1.88 (1.40 to 2.51)] and aspirin ≥150 mg once daily [OR 1.61 (1.25 to 2.07)] were both inferior to warfarin (INR 2–3) for prevention of stroke and systemic embolism in patients with AF. Major bleeding in patients receiving warfarin was comparable to patients on anti-platelets. Of the RCTs included in this meta-analysis only 3 trials [AF-ASA-VKACHINA (47), BAFTA (48), and WASPO (49)] selectively investigated the older population, comparing antiplatelet therapy with warfarin. To date, BAFTA is the largest RCT involving older AF patients. The multi-center RCT randomized 973 patients into dose-adjusted warfarin (INR target 2–3) or aspirin 75 mg daily treatment arms. The warfarin group had fewer numbers of strokes [OR 0.52, 95% CI (0.33–0.80)] and the same number of major hemorrhages [0.96, 95% CI (0.53–1.75)] compared to the aspirin group.

In a more recent meta-regression analysis, Bai et al. (50) identified six studies (47–49, 51–53) that specifically looked at warfarin vs. aspirin in the older population (defined as age ≥65 years old). Unsurprisingly, warfarin was superior to aspirin for the prevention of stroke/thromboembolism [RR 0.44, 95%CI (0.24–0.64)] with no significant increase in major bleeding (RR 1.20, 95%CI [0.91–1.50]). With advancing age, the RR for stroke/thromboembolism for warfarin vs. aspirin was attenuated (*r*^2^ = 0.76, *P* = 0.002) but no significant reductions were observed in the RR for major bleeding (*r*^2^ = 0.20, *P* = 0.22).

### Newer Oral Anticoagulants

The NOACs, also referred to as direct oral anticoagulants (DOACs), provide an alternative to warfarin for thromboprophylaxis. They may be more favorable in the older person, given that they do not require routine monitoring, have a wider therapeutic window and have fewer food and drug interactions unlike warfarin (54).

The efficacy and safety of NOACs have been demonstrated in large RCTs comparing these drugs (i.e. dabigatran, rivaroxaban, apixaban and edoxaban) to warfarin (55–58). These are supported by numerous “real world” studies which have also demonstrated these findings. (33, 59). However, in these RCTs, older patients were under-represented with less than half of the study populations comprising of participants ≥75 years of age. Subsequent analyses of the data from these trials (Table 2) demonstrated that NOACs were non-inferior to dose adjusted warfarin for the prevention of stroke and systemic emboli even in the older cohorts (60–63). Despite this, an issue consistently raised by clinicians with respect to these trials is that they are not truly reflective of real-world practice. Study participants are not regarded as accurate depictions of real-world patients, as they represent a minority who are uncomplicated and “fit” enough to fulfill stringent study criteria. Further, many of the systematic reviews and meta-analyses comparing warfarin to NOACs are based on RCT data, with few based on real world studies and focused on older people (Table 3). Nonetheless, these results indicate that NOACs are generally preferable to warfarin for prevention of stroke and systemic emboli in patients aged ≥75 years, while the rates of major bleeding are comparable.

**Table 2:** Secondary analyses studies of the phase III trials of NOACs vs. warfarin.

**Study**	**Population**	**Older patients %**	**Intervention**	**Stroke & SE (95% CI)**	**Major bleeding (95% CI)**
RE-LY (55)	18,113	<75 years: 59.9 75–79 years: 23.4 80–84 years: 12.7 ⩾85 years: 4.0	Dabigatran 110 mg b.i.d Dabigatran 150 mg b.i.d	<75: HR 0.93 (0.70–1.22), 75–79 HR 1.08 (0.73–1.60), 80–84 HR 0.75 (0.46–1.23), ⩾85 HR 0.52 (0.21–1.29) *P* interaction = 0.394 <75 HR 0.63 (0.46–0.86), 75–79 HR 0.65 (0.42–1.01), 80–84 HR 0.67 (0.41–1.10), ⩾85 HR 0.70 (0.31–1.57) *P* interaction = 0.996	<75 HR 0.62 (0.50–0.77), 75–79 HR 0.93 (0.71–1.21), 80–84 HR 1.18 (0.84–1.65), ⩾85 HR 1.01 (0.59–1.73) *P* interaction = 0.006 <75 HR 0.70 (0.57–0.86), 75–79 HR 1.04 (0.81–1.35), 80-84 HR 1.41 (1.02–1.94), ⩾85 HR 1.22 (0.74–2.02) ***P*** **interaction** **=** **0.001**
ROCKET AF (56)	14,264	⩾75 years: 43.7	Rivaroxaban 20 mg daily	<75 HR 0.95 (0.76–1.19), ⩾75 HR 0.80 (0.63–1.02) *P* interaction *P* = 0.313	<75 HR 0.96 (0.78–1.19) ⩾75 HR 1.11 (0.92–1.34) *P* interaction *P* = 0.336
ARISTOTLE (57)	18,201	<65 years: 30.1 65–74 years: 38.7 ⩾75 years: 31.2	Apixaban 5 b.i.d	<65 HR1.16 (0.77–1.73), 65–74 HR 0.72 (0.54–0.96), ⩾75 HR 71 (0.53–0.95). *P* interaction = 0.11	<65 HR 0.78 (0.55–1.11), 65–74 HR 0.71 (0.56–0.89), ⩾ 75 0.64 (0.52–0.79), *P* interaction = 0.63
ENGAGE AF-TIMI 48 (58)	21,105	<65 years: 26.0 65–74 years: 33.8 ⩾75 years: 40.2	Edoxaban 60 mg daily	<65 HR 0.94 (0.65–1.37), 65–74 HR 0.89 (0.68–1.16),⩾75 HR 0.83 (0.66-1.04). *P* interaction = 0.84	<65 HR 0.81 (0.58–1.12), 65–74 HR 0.75 (0.60–0.94),⩾75 HR 0.83 (0.7–0.99). *P* interaction = 0.78

*RE-LY, Randomized evaluation of long-term anticoagulation; ROCKET-AF, Rivaroxaban for stroke prevention in patients with non-valvular atrial fibrillation; ARISTOTLE, Apixaban for reduction in stroke and other thromboembolic events in atrial atrial fibrillation; ENGAGE AF-TIMI 48, Effective Anticoagulation With Factor Xa Next Generation in Atrial Fibrillation-Thrombolysis In Myocardial Infarction; b.i.d, twice daily. All studies used dose adjusted Warfarin INR 2.0–3.0. HR, Hazard Ratio; CI, confidence interval; Bold, significant interaction; SE, systemic embolism*.

**Table 3:** Meta-analysis of NOACs vs. Warfarin for stroke prevention in older individuals with AF.

**References**	**Study design**	**Comparison**	**Stroke/SSE (95%CI)**	**Major hemorrhage (95% CI)**
Bai et al. (50)	SR + MRA (RCT = 7, cohort = 7, national registry = 1 and observational = 1)	NOAC^†^ vs. Warfarin	≥65 years: HR 0.81 (0.73–0.89)	≥65 years: HR 0.87 (0.77–0.97)
Kim et al. (64)	SR + MA (RCT = 5)	NOAC^†^ vs. Warfarin	≥ 75 years: RR 0.83, (0.69–1.00)	equivalent safety^*^
Ruff et al. (65)	MA (RCT = 4)	NOAC^†^ vs. Warfarin	<75 years RR 0·85 (0·73–0·99) ≥75 years RR 0·78 (0·68–0·88)	<75 years RR 0·79 (0·67–0·94) ≥75 years RR 0·93 (0·74–1·17)
Briceno et al. (66)	SR+MA (RCT = 7)	NOAC^†^ vs. Warfarin	≥75 years: OR 0.77 (0.68–0.87)	^*^
Sardar et al. (67)	MA (RCT = 10)	NOAC^‡^ vs. conventional therapy^§^	≥75 years: OR 0.65 (0.48–0.87)	≥75 years: OR 1.02 (0.73–1.43)
Lega et al. (68)	MA (RCT = 3)	NOAC^‡^ vs. Warfarin	<75 years RR 0.83 (0.71–0.96) ≥75 years: RR 0.77 (0.67–0.89)	<75 years RR 0.73 (0.65–0.81) ≥75 years RR 0.90 (0.82–1.00)

SSE, Stroke or systemic embolism; HR, Hazard Ratio; RR, Relative risk; OR, Odds ratio; SR, Systematic review. MA, meta-analysis; MRA, Meta-regression analysis; RCT, Randomized controlled trials;

* Not reported;

† NOACs included were dabigatran, rivaroxaban, apixaban, and edoxaban;

‡ NOACs included dabigatran, rivaroxaban, and apixaban;

§ *conventional therapy included Vitamin K antagonists, low-molecular weight heparin, aspirin, and placebo*.

Given that every NOAC has its own efficacy and safety profile, comparing them collectively as a group against warfarin would not be just, nor would it provide clinicians with an accurate idea of their benefits and risks with real-world use. Lin et al. (69), in their systematic review and network meta-analysis, have shed some light on this. Their study included both RCTs and non-RCTs to provide a real-world assessment of the NOACs. They compared dabigatran, rivaroxaban, apixaban, and edoxaban individually with warfarin for stroke and systemic emboli (SSE) and major hemorrhage. In patients aged ≥75 years, dabigatran 150 mg and apixaban both significantly reduced events of SSE (rate ratio 0.74 and 0.69 respectively, 95% CI <1 for both), whilst other NOACs showed no significant differences to warfarin. In terms of adverse events, only apixaban was associated with a clinically significant reduction in major bleeding in older people [rate ratio 0.67 (95% CI 0.55–0.82)]; all other NOACs were similar to warfarin for major bleeding.

## Bleeding Risk

Like the stroke risk, advancing age is also accompanied with an increased propensity for bleeding and this bleeding risk is a commonly cited reason for OAC underuse and inappropriate cessation (70, 71). OAC cessation leads to worse outcomes amongst older people (72, 73). Indeed, the HAS-BLED scoring system recognizes age as an independent risk factor for bleeding in anticoagulated patients and a score of ≥3 classifies individuals as “high-risk” (Table 4) (74). However, both the NICE and ESC guidelines recommend using the score to identify and treat reversible risk factors associated with this increased risk rather than denying patients of OAC based on the score alone (34, 35).

**Table 4:** Bleeding risk with oral anti-coagulation: HAS-BLED score.

**Characteristic**	**Score**
Uncontrolled Hypertension	1
Abnormal renal and/or liver function	1-2
Stroke history	1
Bleeding	1
Labile INR	1
Age >65 years	1
Drugs and/or alcohol	1-2

*Maximum 9 points. Hypertension defined as systolic >160 mm Hg. Renal disease is dialysis, transplant, creatinine >200 μmol/L. Liver disease is cirrhosis or biochemical evidence of liver derangement (bilirubin >2x normal with AST/ALT/AP >3x normal). Bleeding is either prior major bleeding or predisposition to bleeding (e.g., bleeding diathesis, anemia). Labile INR [international normalized ratios] is unstable/high INR or a time in therapeutic range (TTR) of <60%. Drugs refer to medications predisposing to bleeding (e.g., aspirin, clopidogrel, and NSAIDs]. Alcohol is ≥8 drinks/week*.

The risk of major bleeding associated with warfarin has been explored in a number of reviews; but, the studies included have variable definitions of major bleeding with some considering only extra-cranial bleeding events and others including all bleeding events. Current opinion on the major bleeding risk is mixed as some studies report higher risks with warfarin (75–79), whilst others do not report any significant differences (50, 80, 81).

Nonetheless, all RCTs for NOACs have consistently reported a significantly lower risk of ICH compared to warfarin (55–58). ICH is one of the most feared complications of OAC, particularly in older individuals and can often be a terminal event in this age group. Chao et al. (82), in a nationwide cohort study looked at the use of OAC in very old patients (≥90 years) with AF. They noted that in these nonagenarian, warfarin use was not associated with an increased risk of ICH that was of statistical significance, compared to patients who were not on antithrombotic therapy [HR 1.22 (95% CI 0.68–2.18), *p* = 0.512]. The study also had a second cohort where comparisons were made between warfarin and NOACs. Although stroke risks were similar, the ICH risk was markedly lower with NOACs, indicating that the NCB is higher with these drugs. A similar study by Alnsasra et al. (83) demonstrated a positive NCB favoring OAC therapy in patients over the age of 75 years, with the greatest NCB observed in the group aged 75–84 years, those treated with higher doses of DOACs or with a time in therapeutic range (TTR) of ≥60% (if warfarin treated), highlighting the importance of optimal dosing (84).

NOACs in general, have been shown to have satisfactory safety profiles compared to warfarin in those who are older, with some even demonstrating superiority over the VKA in certain domains (Table 3). Despite this, the lack of a uniform and well-defined guidance for clinicians on the selection of an appropriate OAC/NOAC has resulted in a largely varied practice across the globe, not necessarily dependent on the safety profiles of the drugs. In the initial stages, dabigatran, a drug with a relatively higher risk of overdose and GI bleeding, due to the longer half-life, and predominant renal dependence for clearance, was avidly prescribed. This may have been because dabigatran was the only approved NOAC at one point in time that also had a reversal agent. However, recent evidence has demonstrated declining prescription rates for dabigatran, particularly in older patients (85, 86). Increasing awareness, availability of other NOACs and the development of antidotes for these may be amongst the contributing reasons.

Sharma et al. (87) reviewed the safety and efficacy of NOACs in a large systematic review and meta-analysis that included RCT data on NOACs prescribed for both stroke prevention in AF as well as secondary prevention of venous thromboembolism. Compared to VKAs, apixaban, and edoxaban were associated with significant reductions in major hemorrhage [odds ratio [OR] range 0.63–0.81, 95% CI <1, *p* < 0.05 for both] in the population aged ≥75 years. However, both dabigatran 150 mg (OR 1.78, 95% CI 1.35–2.35), and 110 mg (OR 1.40, 95% CI 1.04–1.90) increased the risk of GI bleeding in these patients. Regardless, dabigatran 150 mg (OR 0.43, 95% CI 0.26–0.72), and 110 mg (OR 0.36, 95% CI 0.22–0.61) still had significantly lower risks of ICH when compared to VKAs. These observations have been replicated in a meta-analysis of real-world studies by Romanelli et al. (88) where both dabigatran 150 and 110 mg were associated with a significantly lower risk of ICH compared to warfarin. However, patients aged ≥75 years had an ~50% higher chance of gastrointestinal bleeding compared to younger patients. The findings warrant caution when prescribing high dose dabigatran for older individuals, who have multiple concomitant risk factors for GI bleeding.

The analysis of phase III trial data also showed that rivaroxaban is non-inferior to warfarin and is not associated with significant major bleeding (Table 2). However, significantly higher rates of the combined end point of major bleeding and clinically relevant non-major bleeding (*P* interaction = 0.009) were observed in older patients taking rivaroxaban. This was thought to be driven primarily by gastrointestinal bleeding. However, analysis of NCB based on prevention of ischaemic stroke, life threatening bleeding and all-cause mortality indicated that the overall benefit of rivaroxaban over warfarin was still higher in the older cohort (60).

In a sub-analysis of the ARISTOTLE trial, apixaban was associated with lower rates of major bleeding (HR 0.66, 95% CI 0.48–0.90), and ICH (HR 0.36, 95% CI 0.17–0.77) in patients ≥80 years of age, relative to warfarin. In the AVERROES trial which compared apixaban and aspirin, rates of major bleeding were similar for ≥75 and ≥85 age groups (89). Further, the ENGAGE AF-TIMI 48 trial also suggested a greater NCB with edoxaban with advancing age. The *post-hoc* analysis demonstrated that the benefits were maintained even in the extremes of age (≥80 and ≥85 years) (62). Again, this was primarily thought to be due to the reduction in major bleeding and ICH associated with edoxaban.

Understandably the decision to initiate OAC in older patients and the choice of anticoagulant, can pose a dilemma for clinicians but often, this can be driven by patients' risk factor profiles and preferences. A study by Devereaux et al. (90) showed that compared to physicians, patients placed more value on avoiding strokes rather than avoiding bleeding. The authors strongly recommended giving importance to what patients considered significant during the decision-making process. Some may favor stroke prevention and accept the bleeding risk whilst others, may choose not to take a medication that may increase their risk of bleeding.

The lack of direct comparisons between the NOACs and the heterogeneity in patient populations between the phase III trials add to the challenge of deriving clear guidelines for NOAC use in older patients. Hence, the choice of NOAC should be based on a comprehensive review of comorbidities such as renal dysfunction and other variables such as cognitive decline, nutrition status, and polypharmacy.

## Frailty

Frailty is described as a clinically recognizable state of increased vulnerability due to an age-associated decline in reserve and function across multiple physiological systems, compromising an individual's ability to cope with daily activities or acute stressors (91). The growing understanding of its association with the risk of adverse outcomes such as falls, worsening disability, hospitalisations and mortality has led to a significant focus on this syndrome (92). Frailty is thought to be reflective of an individual's biological age; a more accurate predictor for the occurrence of such adversities compared to chronological age. That said, it is more prevalent in those who are >65 years, and females in particular (93, 94).

While a quarter to three quarters of people over the age of 85 years is thought to be frail, up to three quarters may also not be frail, suggesting that there are significant variations in the way frailty develops, is assessed, and classed (95). Data on the prevalence of frailty in patients with AF is limited in the literature, highlighting a lack of research in this field. A recent systematic review of 11 studies by Villani et al. (93) focusing on this very subject, identified a frailty prevalence ranging from 4.4 to 75.4%. Age was regarded as the principle factor for the large variation, given the low prevalence of 4.4% reported in the study by Ng et al. (96), where the mean age of the participants was lower at 66.7 ± 7.8 years. However, it is worth noting that the majority of the included studies were based on hospitalized in-patients whose general health is likely to have been poorer. In another meta-analysis by Wilkinson et al. (97), frailty prevalence in AF patients varied from about 6% in a registry of outpatients to about 100% in a nursing home population. This suggests that the numbers quoted in studies are largely dependent on the study setting and likely overestimated as most are hospital based. Further, the criteria for assessing frailty differed between the studies, preventing direct comparisons. Hence, the true prevalence of frailty in the general AF population is yet to be determined and longitudinal studies where frailty assessment is based on more homogeneous criteria are needed (93). Like many factors, frailty status is dynamic and subject to change over time.

There are a variety of tools used in the identification, assessment and gradation of frailty. Broadly speaking, the concept of frailty is based on two main models (Table 5); the phenotype model (Fried's Criteria) and the cumulative deficits model [Frailty Risk Index/Frailty Index (FI)]. Subsequent tools building on these, such as the Rockwood Clinical Frailty Scale, Edmonton Frail Scale, and the Groningen Frailty Indicator have since developed (99–101).

**Table 5:** Summary of key characteristics of the two frailty models.

**Features**	**Frailty phenotype by Fried et al**.	**Frailty Index by Rockwood et al**.
Definition	Described by physical and functional characteristics	An accumulation of deficits related to activities of daily living
Criteria	Pre-defined/set criteria (of 5 phenotypic components)	Non-fixed set of variables (of at least ≥30)
Criteria assessed	• Unintentional weight loss (of more than 10 lbs in the past year) • Self-reported exhaustion • Weakness (assessed by grip strength) • Slow walking speed • Low physical activity	Can be developed based on any symptom, sign, abnormal laboratory value, disease classification, or disability that:• Is present in at least 1% of the population • Covers several organ systems • Has no more than 5% missing data
Variables	Categorical	Continuous or Categorical
Frailty concept	Frailty is present/absent	Frailty is gradable
Frailty grading	0-Robust1-2–Pre-frail3-Frail	**Continuous:** Number between 0 and 1, where the greater the number, the more frail a person isCategorical:^*^ ≤ 0.03–Relatively fit >0.031– ≤ 0.10–Less fit >0.10– ≤ 0.21–Least fit >0.21– ≤ 0.45–Frail ≥0.45–Most frail
Comprehensive geriatric assessment	Not required	Required
Cognition	Does not consider cognitive impairment	Considers cognitive impairment
Co-morbidities	Does not consider co-morbidities	Considers co-morbidities such as dementia
Prognostication	Predictive of adverse outcomes	Predictive of adverse outcomes
Relationship to disability	Disability is seen as a result of frailty.	No clear distinction between disability and frailty. Disabilities contribute to frailty.
Applicability	Not applicable to patients with disabilities More suitable for immediate identification of frail/ at risk individuals	Applicable to patients with disabilities Can be used in the long-term follow-up of an individual
Limitations	Does not include psychosocial components of frailty Cannot be used on patients with mental/physical limitations Certain components (e.g., grip strength) are not routinely done in clinical practice	Time consuming to calculate Comparison of results between different institutions may not be possible if different variables are measured/ assessed

* *Cut-off points proposed in study by Song et al. (98)*.

The phenotype model, based on physical and functional indicators, was developed by Fried et al. (102) using data from the Cardiovascular Health Study (CHS), performed on a community-dwelling population. The landmark study identified comorbidity as a risk factor for, and disability as a consequence of frailty, describing an overlap but distinguishing between the three. Five pre-defined criteria which include unintentional weight loss (of more than 10 lbs in the past year), self-reported exhaustion, weakness (assessed by grip strength), slow walking speed and low physical activity are assessed and a point is allocated for each variable if present. A score of 0, 1–2 and 3 represent individuals who are robust, at risk of developing frailty (pre-frail) and frail, respectively. Although this model enables assessment of the frailty status in individuals without the need for a comprehensive clinical assessment, it does not offer any information about the underlying conditions which may be contributing to the presence of these variables. Further, the tasks though simple at first glance, may be challenging to execute in the presence of certain physical and cognitive limitations, and in the absence of equipment such as hand-held dynamometers that are not readily available in the clinical setting.

The cumulative-deficits model in contrast, was developed from the Canadian Study of Health and Aging (CSHA) and assumes that frailty is a result of an accumulation of individual deficits (inclusive of symptoms, signs, abnormal laboratory values, disease classifications, and disabilities) (103, 104). A comprehensive geriatric assessment is required and each variable whether a hearing impairment or a diagnosis of atrial fibrillation is presumed to have an equal effect on the frailty status and risk of adverse outcomes. The FI is derived from a mathematical calculation where the number of individual “deficits” possessed by an individual is divided by the total number of variables or deficits measured. For example, if 10 of 20 variables are present, the FI is 10/20 = 0.5. The number derived may be used as a continuous variable (i.e. the higher the number, the more likely an individual is to be frail and the less their physiological reserve will be, to adapt to stressors and allow accumulation of further deficits) (103, 105).

The FI is particularly advantageous in the long-term follow-up of a patient as changes to this number highlight improvements or worsening of the frailty status. This also emphasizes the notion that frailty is gradable rather than being present/absent (105). The FI has also been categorized using pre-defined cut-off points by Rockwood et al. which have varied in studies. In one of their studies, classifications were made as follows: ≤ 0.03–relatively fit, >0.031– ≤ 0.10–less fit, >0.10– ≤ 0.21–least fit, >0.21– ≤ 0.45–frail, and ≥0.45–most frail and in another, the FI cut-off for frailty was ≥0.25 (98, 106). Although the original study involved assessment of 92 different parameters, subsequent research has indicated that this can be reduced to a more feasible number of about 30, without loss of predictive value (103). Thus, items included in the FI are not fixed and any variable (that fulfills specified criteria) can be incorporated. While this allows flexibility, the lack of a standardized “proforma” and categorisations that translate into classes of risk pose issues in clinical practice as well as research.

Despite the unique features between the phenotype and cumulative deficits models, a significant overlap exists in their ability to predict future adverse health outcomes such as falls and death (107). Their applicability and usefulness however, may depend on the general state of an individual at the time of evaluation. The correct and combined use of both models is therefore recommended as they provide complementary information on the individuals and their risk factor profiles (105). However, the exhaustive nature of the models limits their use as quick and convenient bed-side screening tools that can be used easily by clinicians. Currently, comprehensive geriatric assessments are considered the “gold-standard” for frailty assessment (108).

Frailty is extremely relevant to the older AF population as it is one of the most commonly cited reasons for OAC under-prescription when in fact, frail patients may stand to achieve the most benefit from it (109, 110). In a recent systematic review and meta-analysis, Oqab et al. (111) demonstrated that frailty significantly reduced the prescription of OAC (OR 0.49, 95% CI 0.32–0.74), with cognitive impairment and malnutrition amongst the other commonly cited geriatric reasons. One explanation for this is the association between frailty and falls and the consequent risk of ICH.

According to a meta-analysis by Cheng et al. (112) frail older adults are at a higher risk of having recurrent falls compared to the pre-frail and robust older adults groups. Whilst this may be an irrevocable fact, it is important to consider the individual factors contributing to the individual's frailty status and falls risk. Frailty does not equate to having falls and often, the risk of falls can correspond to potentially reversible factors and be minimized by addressing these. This includes provision of visual and hearing aids for those with sensory impairments, performance of mobility assessments on at-risk patients such as those with gait impediments, provision of mobility aids and balance-improving exercises for patients who require them and adjustments to the general area of living where needed (113).

To further evaluate the OAC-associated bleeding risk in AF patients who were at risk of developing falls, Man-Son-Hing et al. (114) designed a Markov decision analytic model and performed multiple analyses. In the base-case analysis which included older individuals with an average annual stroke risk of 6% and falls risk of 33%, warfarin was associated with the highest quality-adjusted life expectancy compared to aspirin or no treatment at all. Warfarin remained the superior choice, regardless of the annual risk (from 0 to 100%) of having a fall. The study also estimated that an older patient taking warfarin would need to fall 295 times a year to offset the benefits of OAC. This suggests that the perceived falls-related ICH risk is higher than the actual risk, owing to an increased, perhaps exaggerated fear surrounding OAC prescriptions in older individuals. The authors proposed that this may be due to the significance of the event which clinicians are more likely to remember, despite the rarity.

Despite a risk of falls, patients can continue to derive benefits from being anticoagulated, the key one being prevention of stroke. Thus, falls or risk of it alone should not be absolute contraindications to OAC (114). In an observational study of hospitalized frail, older patients, those who were non-anticoagulated had significantly more events of ischaemic stroke and clinically relevant bleeding after 1 year follow up, compared to patients who were on OAC. However, no statistical difference was observed in the rates of re-hospitalization, mortality and falls between the anticoagulated, and non-anticoagulated groups (110). The increased bleeding events in the non-anticoagulated group may have been a reflection of their higher baseline bleeding risk that may have led to an aversion from anticoagulation in the first place.

In contrast, Papakonstantinou et al. (115) demonstrated a higher all-cause mortality in non-anticoagulated old, frail patients after 1 year of follow up. Patients discharged without OAC had higher HEMORR_2_HAGES (Hepatic or renal disease, Ethanol abuse, Malignancy, Older age, Reduced platelet count or function, Re-bleeding, Hypertension, Anemia, Genetic factors, Excessive fall risk, and Stroke), and clinical frailty scale (CFS) scores and a lower Katz score indicative of an increased bleeding risk, greater degree of frailty, and poorer functional status, respectively. However, the study was small, and although these findings were observed in the group without anticoagulation, it was not clear whether these were the reasons that prevented OAC prescription and if the increased mortality was attributable to AF alone.

Studies looking into OAC prescription rates in frail people have been variable. While practices in countries such as Australia and Mexico have shown that frailty status predicted OAC use in older adults (109, 116) there is also evidence to suggest a lack of association between the two (117, 118). This is likely a reflection of the variations in practice and guideline recommendations between the regions as well as the perception of frailty by those assessing it. A sub-study from the ORBIT-AF registry (119) revealed that patients with cognitive impairment and frailty were both less likely to be prescribed OAC when in fact, they had a higher predicted risk of stroke and observed mortality. Moreover, the multivariable analysis demonstrated that there were no interactions between OAC use and frailty, in their associations with mortality, major bleeding, and the composite end point of stroke, systemic embolism, TIA, myocardial infarction, and cardiovascular death.

Nevertheless, the OAC prescription rate of 70% observed in the FRAIL-AF study (120) compared to rates of 35–65% seen in historical studies was encouraging, as it suggested a more judicious use of OAC in older patients, particularly as a significant proportion in the study had diagnoses such as dementia. However, the authors noted that non-frail to moderately frail patients were 3.5 times more likely to receive OAC than severely frail patients, irrespective of their thromboembolic and bleeding risk, highlighting that the *impression* of severe frailty significantly influenced OAC prescription decisions. This emphasizes an important point, which is the absence of a standard for assessing frailty and its severity, even with the availability of a vast number of frailty assessment tools, as alluded to before. Adjudication of the degree of frailty is thereby left at the physicians' discretion, often influenced by their own knowledge and experience or a lack thereof. For many, the perception of frailty is accompanied by a sentiment of futility towards prescribing OAC; another key issue that underlies OAC under-prescription and its consequences.

The rising prevalence of frailty necessitates the establishment of a simple and robust frailty assessment tool, applicable to all patients and clinicians. More and more patients with AF are seen outside cardiac settings and similarly, the majority of patients seen in cardiovascular settings comprise older, possibly frail individuals. Hence, the relevance of frailty assessments is no longer limited to geriatricians (or cardiologists) and a validated frailty assessment tool that can be easily utilized for risk stratification is needed to guide decision-making regarding OAC (94). Indeed, presence of frailty should not be the sole determinant and for this reason, having a pre-set cut-off beyond which OAC would not be prescribed may be unwise, as it may lead to an unjustified preclusion of treatment and act as a barrier to an individualized treatment approach. Denying OAC in a frail patient may achieve nothing but a debilitating stroke that adds to their frailty and burden.

ESC guidelines for AF management include a brief section on frail and “elderly” patients which discusses limited available evidence but no recommendations are made with regards to OAC therapy (34). Addressing this with consensus guidelines will undoubtedly benefit patients and physicians.

## Cognitive Impairment

Several studies have shown an increased incidence of cognitive decline and dementia, including Alzheimer's and vascular dementia, in patients with AF (121–123). Dementia and AF share common cardiovascular risk factors such as heart failure, hypertension, excessive alcohol intake, smoking and diabetes mellitus that could account for this link (124). Regardless, longitudinal studies adjusted for these comorbidities have still shown an association between AF and cognitive impairment (121, 125).

A number of mechanisms have been proposed, and the most familiar concept is the thromboembolic phenomenon which clinically presents as a stroke, resulting in cerebral infarction. However, cognitive decline has been observed even in the absence of strokes and small vessel disease and/or cerebral microinfarcts have been identified as an underlying cause (126). These “silent” strokes have no clinical manifestation, only apparent on neuroimaging. AF is considered an independent risk factor for cerebral microinfarcts (127).

Another proposed explanation for the link between AF and cognitive impairment is the beat-to-beat variability that occurs in AF, leading to intermittent cerebral hypoperfusion. This in turn results in cerebral ischaemia, particularly in the white-matter regions, and is regarded as a frequent mechanism for cognitive impairment in AF (128, 129). Other inflammatory and neurohormonal processes are also likely contributory (127).

The meta-analysis by Kalantarian et al. (130) highlighted a significant association between AF and dementia, independent of stroke. In the Atherosclerosis Risk in Communities Neurocognitive (ARIC-NCS) study (131) which followed 12,515 patients for over 20 years, incident AF was associated with a 23% higher risk of dementia, even after adjustments were made for cardio- and cerebrovascular risk factors as well as prevalent and incident ischaemic stroke. The mean age of the participants was 56.9 ± 5.7 and although the incidence rates of dementia were significant in those under and over the age of 57 years, it was more pronounced in the >57 years group. In contrast, the Rotterdam study (132) which also prospectively followed 6,514 individuals for a similar length of time showed that the risk of dementia was confined only to the younger participants with incident AF, who were <67 years. Further, there appeared to be a dose-response relationship as those who had AF for a longer duration were more likely to develop dementia. This is plausible as dementia develops over many years and AF needs to develop at a younger age to contribute to the onset of this condition at a later stage.

The association between AF and dementia has fuelled a vast amount of research to determine if OACs have any therapeutic benefit in attenuating this risk. Given that OACs largely address the thromboembolic complications when taken appropriately, this should theoretically be the case, though cognitive impairment arising from hypoperfusion and related mechanisms would remain unaffected (127). Existing evidence relating to this subject is non-conclusive with some studies demonstrating a reduction in the incidence of dementia with OACs (133, 134) and others suggesting otherwise (135, 136) (Table 6). The studies where no improvements were observed with OAC were mainly warfarin-based and the quality of anti-coagulation control, that is less likely to be achieved with VKAs such as warfarin may explain this observation. Cheng et al. (133) in a meta-analysis recognized that TTR appeared to play an important role in cognitive benefit as TTR <25% significantly increased risk of dementia compared to TTR ≥ 75% (HR 3.02, 95% CI 1.12–8.91; *P* = 0.03). Non-adherence, polypharmacy, comorbidities, and drug interactions can all have an impact on TTR in older people but are less likely to influence the quality of anticoagulation with NOACs.

**Table 6:** Key findings from studies reporting on oral anticoagulation and cognitive impairment in AF.

**References**	**Design**	**Population**	**Age (±SD)**	**Main results**
Friberg et al. (45)	Retrospective cohort	444,106	74–81	After propensity score matching, no difference in the dementia risk was observed between NOACs and VKAs (HR 0.97, 95% CI 0.67–1.40)
Mongkhon et al. (134)	Meta-analysis	452,878	71–81	OAC use reduced dementia risk compared to no OAC use (RR 0.79, 95% CI 0.67–0.93) A higher percentage of TTR significantly reduced dementia risk (RR 0.38, 95% CI 0.22–0.64)
Cheng et al. (133)	Meta-analysis	471,057	> 63	OACs reduced cognitive impairment in patients with AF (HR 0.71, 95% CI 69–0.74; *P* < 0.00001) NOACs were better than warfarin in terms of the protective effect on cognition (HR 0.51, 95% CI 0.37–0.71; *P* < 0.00001)
Moffitt et al. (136)	Meta-analysis	18,876	–	No definitive evidence of cognitive benefit or harm from anticoagulation
Mavaddat (135)	RCT	973	81.5 ± 4.3	No evidence that anticoagulation protects against cognitive decline
Madhavan et al. (119)	Observational	2,800	71.2	OAC with warfarin was associated with an ~22% lower risk of dementia (HR 0.78, 95% CI 0.64–0.97)
Jacobs et al. (137)	Retrospective cohort	5,254	72.4 ± 10.9	Compared to warfarin, patients on NOACs were 43% less likely to develop stroke/TIA/dementia (HR 0.57, 95% CI 0.17–1.97; *p* = 0.38)
Bunch et al. (138)	Retrospective cohort	10,537	69.3 ± 10.9	Low TTR increased risk of dementia in AF (HR = 2.51, *P* = 0.005)
Barber et al. (139)	Observational	258	72	Warfarin use compared to aspirin, was associated with reduced prevalence of dementia (18 vs. 32% *P* = 0.023)

*OAC, oral anticoagulants; HR, hazard ratio; NOACSs, novel anticoagulants; TIA, transient ischaemic attack; TTR, time therapeutic range; AF, atrial fibrillation; RCT, Randomized Controlled Trial, RR, risk ratio; CI, confidence interval; VKA, vitamin-K antagonist*.

Recent evidence suggests that NOACs may be preferable for reducing incidence of dementia (137). They may also improve adherence as they can be given as once or twice daily regimens without the need for frequent follow-up and can easily be incorporated into multi-drug regimens as well as blister packs and dosette boxes, in patients with cognitive impairment. However, opinions are mixed on whether NOACs or VKAs are better for adherence, in the context of cognitive impairment (140, 141). Persistence with therapy is also a challenge, and efforts to improve persistence and adherence are needed (142, 143).

On balance OACs, particularly NOACs, appear to be beneficial for reducing the risk of dementia, owing to the reduction in silent cerebral ischaemia or microemboli. However, well-designed RCTs adjusting for confounders, with a longer duration of follow up are awaited to fully explore the impact of OAC on cognitive function (144).

Despite the established benefits of OAC, cognitive impairment remains another reported reason precluding OAC use, over fears of a drug overdose or falls which can result in a bleed. Most individuals with cognitive impairment have their medications administered and are under substantial supervision either by their family members at home or carers in long-term care facilities. In such cases, the likelihood of overdosing on an OAC or incurring harm due to an environmental hazard is low and withholding OAC would only increase the chances of developing a stroke which can cause a greater disability. Of course, there are always more complex cases and in such situations, it may be prudent to have multi-discliplinary discussions to determine the best course of action.

The risk of falls and dementia are addressed in current ESC guidelines which advise withholding OAC only in patients who are likely to have severe uncontrollable falls (e.g., epilepsy) or in selected patients with dementia where caregivers cannot ensure compliance and treatment adherence (34).

## Rate vs. Rhythm Control in Older People

Current NICE guidelines in the UK, recommend that clinicians consider rate control as first line except in the following cases: AF has a reversible cause, new-onset AF, heart failure caused by AF, patients with atrial flutter suitable for ablation and cases where rhythm control is more suitable based on clinical judgement (35). The ESC in their guidance suggests that rhythm control may be indicated in patients who remain symptomatic despite adequate rate control therapy. ESC maintains that the evidence for both rhythm control and rate control is fairly balanced but recognize that data regarding modern rhythm control strategies such as catheter ablation, combination therapy, and early treatment is awaited (34). American guidelines are also on par and outline situations where rhythm control strategies may be attempted and this includes a younger, rather than older patient age (36).

Given that AF is an abnormality of cardiac rhythm, it is reasonable to assume that rhythm control is favorable over rate control. However, in a sub-analysis of the AFFIRM (Atrial Fibrillation Follow-up Investigation of Rhythm Management) Trial (145) all-cause mortality was significantly lower in the rate controlled group who were between 70 and 80 years old, compared to the respective rhythm control group (HR 0.77, 95% CI0.63–0.94, *p* = 0.01). All-cause hospitalization was also lower in this group (HR 0.76, 95% CI 0.68–0.86; *P* < 0.001). There were no differences in the incidence of stroke or major bleeding between the two arms.

An ancillary analysis of the REPOSI study (146), a multi-center observational registry, did not demonstrate significant differences in the cardiovascular and all-cause mortality of older patients managed using rate and rhythm control strategies. In the study, the rate control group was older and had higher rates of polypharmacy, heart failure, and diabetes. Conversely, patients on rhythm control were younger with fewer comorbidities and a better cognitive status. 83% of patients in the study were managed using rate control strategies, indicating that this is the preferred method. This could be because they are viewed as more conservative and less burdensome on older people, and particularly as it makes no difference to the OAC status as its continuation is still recommended due to the high risk of AF recurrence.

Rate control vs. rhythm control strategies for AF continue to be debated in the literature; however there is paucity of data with respect to those who are of an older age. Whilst some studies suggest that rate control strategies are superior in terms of cost-effectiveness (147), others have noted better outcomes and health-related quality of life with rhythm control interventions (148, 149). Nonetheless, real world studies indicate that rate control is more favored and the vast majority of older patients are managed this way (41).

## Conclusion

AF is a significant global health burden that is more prevalent in older people. Management of this condition in the geriatric population is riddled with clinical dilemmas. They are accompanied by a high thromboembolic risk but also a concomitant high bleeding risk, requiring clinicians to balance the NCB (150). Indeed, these require deliberation to prevent harm to patients but often, clinical situations are complicated by the under-estimation of the thromboembolic risk and over-estimation of the bleeding risk. Consequently, OACs continue to be under-used in older individuals.

When making decisions regarding OAC, chronological age is of less importance and biological age, indicated by an individual's frailty and functional status as well as factors such as cognitive impairment must be given careful attention. Nevertheless, their presence should not be regarded as absolute contraindications to OAC use. Risk factor modification, identification of barriers to treatment and involvement of patients and their family members are crucial to the initiation of OAC and improvement of treatment adherence. In the absence of contraindications and as allowed by patients' risk factor profiles, clinicians should explore NOACs as an alternative to conventional VKAs, particularly as they have been shown to reduce the risk of ICH.

OAC prescription practice in older individuals largely varies between regions and even from physician to physician within the same locality. While some guidelines are starting to address certain difficulties faced in treating old patients with OAC, further improvements are needed including consensus recommendations, although the challenges to this must be appreciated.

With the aging population and the anticipated rise in AF prevalence, it is imperative that regulatory bodies and clinicians take responsibility to ensure patients with this condition are treated appropriately and holistically. Indeed, there is a move toward a more integrated or holistic approach to AF management that can be summed up as follows: “A” Avoid stroke; “B” Better symptom control with symptom directed decisions on rate or rhythm control; “C” Cardiovascular risk, and comorbidity management, including attention to lifestyle changes (151). Such an integrated approach has been associated with improved outcomes and reduced healthcare costs (152–154).

## Author Contributions

ZZ, AK, and AF performed the literature search, wrote the manuscript, and constructed the tables. This was supervised by GL who revised and approved the final version of the manuscript.

### Conflict of Interest Statement

GL Consultant for Bayer/Janssen, BMS/Pfizer, Medtronic, Boehringer Ingelheim, Novartis, Verseon, and Daiichi-Sankyo. Speaker for Bayer, BMS/Pfizer, Medtronic, Boehringer Ingelheim, and Daiichi-Sankyo. The remaining authors declare that the research was conducted in the absence of any commercial or financial relationships that could be construed as a potential conflict of interest.
